# Fluorescently tagged Lin7c is a dynamic marker for polarity maturation in the zebrafish retinal epithelium

**DOI:** 10.1242/bio.20135371

**Published:** 2013-07-01

**Authors:** Marta Luz, Elisabeth Knust

**Affiliations:** Max-Planck-Institute of Molecular Cell Biology and Genetics, Pfotenhauerstrasse 108, D-01307 Dresden, Germany

**Keywords:** Polarity, Neuroepithelia, Retina, Crumbs, Zebrafish

## Abstract

Development of epithelial cell polarity is a highly dynamic process, and often established by the sequential recruitment of conserved protein complexes, such as the Par or the Crumbs (Crb) complex. However, detailed insights into the refinement of polarity and the formation of the complexes are still lacking. Here, we established fluorescently tagged Lin7c, a core member of the Crb complex, as an ideal tool to follow development of polarity in zebrafish epithelia. We find that in gastrula stages, RFP-Lin7c is found in the cytosol of the enveloping layer, while Pard3-GFP is already polarized at this stage. During development of the retinal epithelium, RFP-Lin7c localization is refined from being cytosolic at 14 hours post fertilization (hpf) to almost entirely apical in cells of the eye cup at 28 hpf. This apical Lin7c localization depends on the Crb complex members Oko meduzy and Nagie oko. Thus, fluorescently tagged Lin7c can be used in a broad range of epithelia to follow polarity maturation in vivo and specifically to elucidate the sequence of events determining Crb complex-mediated polarity.

## Introduction

Epithelial tissues are built from cells with marked polarity, manifested by the polarised cell shape and the uneven distribution of organelles and molecules. The plasma membrane itself is compartmentalised into two distinct regions, the apical domain facing the external environment or a lumen, and the baso-lateral domain, which is in contact with neighbouring cells or a basal lamina. Epithelial cell polarity is crucial for development and maintenance of tissue integrity in numerous developmental processes, such as neurulation or organogenesis, but also in homeostatic processes in differentiated adult tissues. Loss of polarity is associated with various diseases and is considered both a hallmark and precondition for human cancer (reviewed by Laprise, 2011; St Johnston and Sanson, 2011; Ellenbroek et al., 2012; Lim and Thiery, 2012; Martin-Belmonte and Perez-Moreno, 2012).

Several evolutionarily conserved protein complexes regulate establishment and maintenance of epithelial cell polarity, with the Par-3 (Pard3 in zebrafish) and the Crumbs (Crb) protein complexes controlling polarity from the apical pole (Goldstein and Macara, 2007; Assémat et al., 2008; Nance and Zallen, 2011; Tepass, 2012; Roignot et al., 2013). *Drosophila crb* and its vertebrate orthologues are key regulators of apico-basal polarity, both in epithelia and in epithelial-derived photoreceptor cells (PRCs) (Bazellieres et al., 2009; Bulgakova and Knust, 2009). Crb is a conserved transmembrane protein, which forms a membrane-associated protein complex at the apical pole by recruiting the MAGUK (**m**embrane-**a**ssociated **gu**anylate **k**inase) protein Mpp5/Pals1 (zebrafish Nagie Oko (Nok), *Drosophila* Stardust (Sdt)), the multi-PDZ (**P**SD-95/**D**lg/**Z**O-1)-domain protein Patj, and Lin7, which, together with Crb, are conserved from nematodes to mammals and form the core components of the Crb complex (Bulgakova and Knust, 2009; Tepass, 2012). Lin7 is a small scaffolding protein, containing an N-terminal L27 (Lin2/Lin7)-domain and a C-terminal PDZ-domain (Borg et al., 1998; Butz et al., 1998; Kaech et al., 1998; Jo et al., 1999; Bachmann et al., 2004). As in mammals, three *lin7* genes (*lin7a*, *lin7b* and *lin7c*) have been identified in zebrafish (Wei et al., 2006). Although all three are expressed in the eye, Lin7c is the predominant form expressed at early stages in the eye and the developing neural tube (Wei et al., 2006; Yang et al., 2009), therefore we focused our attention to this isoform.

Although the knowledge on the molecules and protein complexes that regulate epithelial cell polarity has increased over the last years, data showing how polarity is initiated and stabilised during development and how polarity complexes are built are scarce. From studies in different model organisms it is clear that development of polarity is a gradual process, which can be regulated by a functional hierarchy of protein complexes. This hierarchy results in a sequential recruitment of polarity complexes to the apical side, as it has been shown for the Par-3 and the Crb complex e.g. during the formation of the blastoderm, the first epithelium in the *Drosophila* embryo (Krahn et al., 2010), or in the follicle epithelium (Morais-de-Sá et al., 2010). Loss of *Drosophila* Crb prevents the apical accumulation of *D*Lin-7 and induces its distribution in the cytoplasm (Bachmann et al., 2008). While our knowledge on the development of polarity in *Drosophila* is quite advanced the dynamics of this process in vertebrate organisms is still largely unexplored due to the lack of appropriate tools.

We sought to analyse the development of polarity in vivo by visualising the sub-cellular distribution of fluorescently tagged Lin7c at different developmental stages of the zebrafish embryo. In comparison to Oko meduzy (Ome), the orthologue of *Drosophila* Crb, and Nok, the small size of the Lin7 molecule makes it an ideal tool as a read-out for the localization of the Crb complex in vivo. We find that during the development of the retinal epithelium Lin7c changes its sub-cellular distribution from entirely cytosolic at early stages to membrane-associated at the apical pole later on, while Pard3 is already found apically at early stages. In addition, we demonstrate that *ome* and *nok* are required for Lin7c apical localisation. Thus, fluorescently tagged Lin7c localization reflects the tissue- and stage-specific maturation of epithelial polarity during development and can be used as an excellent tool to follow developmental changes in Crb-mediated cell polarity in vivo.

## Results and Discussion

### RFP-Lin7c is apically localized in neuroepithelial cells

**Fig. 1.**
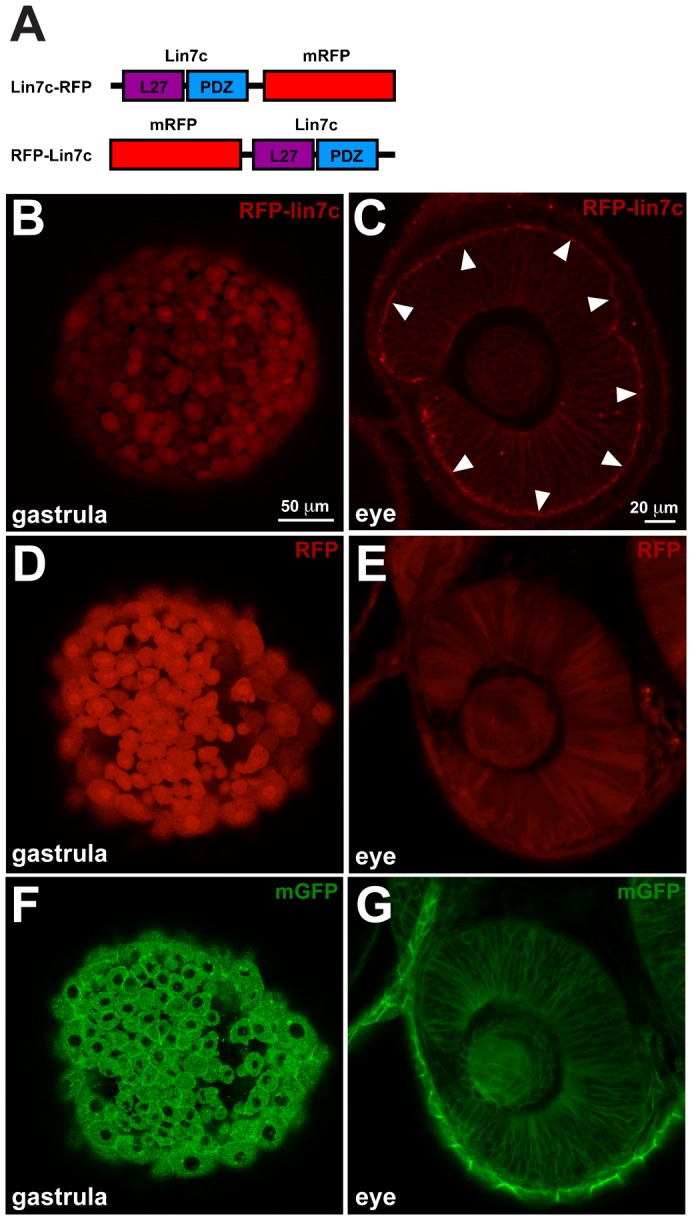
Lin7 sub-cellular localization in neuroepithelial cells is distinct from gastrula cells. Confocal live imaging of RFP-Lin7 sub-cellular localization in zebrafish embryos. (**A**) Schematic representation of Lin7 fusion proteins with monomeric RFP (mRFP). (**B**,**D**,**F**) In gastrula embryos (7 hpf, 70% epiboly stage) RFP-Lin7c, RFP and mGFP are distributed homogeneously in the cells. RFP-Lin7c and RFP are also detected in the nucleus. Scale bar: 50 µm. (**C**,**E**,**G**) Retinal neuroepithelium at 28 hpf. RFP-Lin7c localizes to the cell membranes and is particularly enriched at the apical surface (arrowheads, C). RFP is distributed homogeneously in the cytosol (E) and mGFP localizes to the cell membranes, but is not enriched at the apical surface (G). Scale bar: 20 µm.

To visualize Lin7 protein in live embryos, we tagged Lin7c either at the N- or the C-terminus with monomeric RFP (RFP-Lin7c and Lin7c-RFP, respectively) as schematized in Fig. 1A. To determine the sub-cellular localization of Lin7 in the developing zebrafish embryo, we injected RNA synthesized from RFP-Lin7c-encoding plasmids into one-cell stage embryos and analyzed the distribution of RFP-Lin7c in otherwise wild-type (wt) embryos at different developmental stages using confocal imaging. We used gastrula cells as an example of non-polarized cells and retinal neuroepithelial cells as an example of polarized cells.

During gastrulation stages (7 hpf, 70% epiboly), RFP-Lin7c is localised both in the cytosol and nucleus (Fig. 1B). At 28 hpf, when the eyecup is well formed, RFP-Lin7c is predominantly associated with the membrane and shows a striking accumulation at the apical side in the retinal epithelium (Fig. 1C), a result that is in agreement with the apical localisation of endogenous Lin7 protein (Wei et al., 2006). Lin7c-RFP shows an identical sub-cellular distribution as RFP-Lin7c in both gastrula cells and the retinal epithelium (data not shown). Expression of RFP alone results in a similar distribution as RFP-lin7c in gastrula cells (Fig. 1D), but unlike RFP-Lin7c, RFP does not change its sub-cellular localisation in retinal neuroepithelial cells, where it stays evenly distributed (Fig. 1E). Given the fact that RFP alone shows nuclear localisation and that the RFP-Lin7c fusion protein is relatively small, it is likely that the RFP-Lin7c nuclear localisation results from passive diffusion rather than active nuclear import. A transgenic line expressing a membrane-bound GFP (mGFP) shows mGFP in the plasma membrane and in internal membranes in gastrula cells (Fig. 1F). In retinal neuroepithelial cells mGFP localizes evenly on all cell membranes with no particular enrichment at the apical surface (Fig. 1G). These results show that the sub-cellular distribution of RFP-Lin7c is developmentally regulated, in that it is cytosolic in gastrula cells, while it is associated with the apical membrane in retinal neuropithelial cells.

Importantly, we did not detect any aberrant phenotype in the developing retina upon expression of RFP-Lin7c until 42 hpf. This is in line with results from studies in *Drosophila* and rat, showing that overexpression of Lin7 does not cause any mutant phenotypes nor influence the localization of the other members of the Crb complex (Bachmann et al., 2004; Bachmann et al., 2008; Srinivasan et al., 2008). In contrast, expression of a Myc-Lin7c fusion protein in zebrafish has been reported to induce polarity defects in the developing neural tube (Yang et al., 2009). Whether these discrepancies result from the levels of expressed protein, from the different tags used or from cell type-specific responses remains to be determined.

### Comparison of Pard3 and Lin7c subcellular localisation reflects tissue-specific differences in epithelial polarity

**Fig. 2.**
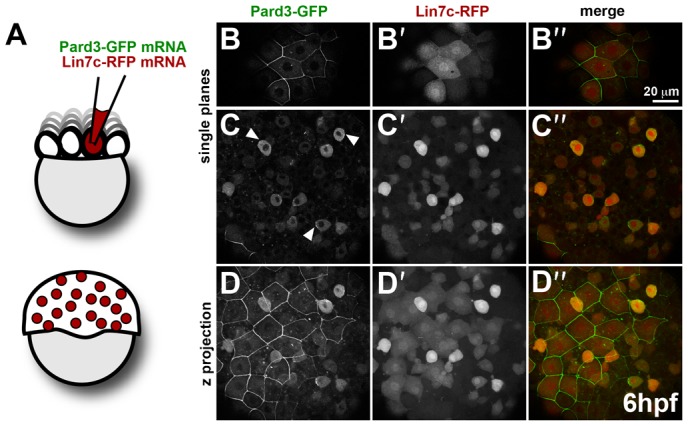
Comparison of Pard3-GFP and Lin7c-RFP subcellular localization reflects tissue-specific differences in epithelial polarity. (**A**) Schematic representation of mRNA injection in 1/16–32 blastomeres for mosaic expression. (**B–D″**) Mosaic co-expression of Par3-GFP and Lin7c-RFP in the gastrula (6 hpf). Par3-GFP is restricted to the membrane of the enveloping layer (EVL) cells (B), while it is mainly cytosolic in the deep cell layer (DEL), with some local accumulations at the cortex (arrowheads) (C). Lin7c-RFP is cytosolic and nuclear both in the EVL (B′) and DEL (C′). (D–D″) Maximum projection of the embryo showing Pard3-GFP (D), Lin7c-RFP (D′) and the merge of both (D″) in the EVL and DEL. Scale bar: 20 µm. Single confocal planes are shown, except for D–D″.

To address whether Lin7 is a polarity marker for other epithelial cells, we analysed its expression during gastrulation. The early zebrafish consists of two embryonic layers, the deep cell layer (DEL) and the enveloping layer (EVL), and the extraembryonic yolk syncytial layer. While the DEL has mesenchymal character, the EVL, the outermost monolayer of cells surrounding the embryo, is a squamous epithelium with well-developed tight junctions (Warga and Kimmel, 1990; Kimmel et al., 1995). We compared the localisation of Lin7c-RFP and Pard3-GFP, a member of the apical Par complex, in these two cell types of the gastrula embryo. To obtain cellular resolution, mosaic expression was achieved by co-injection of mRNA encoding Lin7c-RFP and Pard3-GFP in 1/16–32 blastomeres, as schematized in Fig. 2A. During gastrulation stages, Pard3-GFP is predominantly associated with the plasma membrane in the EVL (Fig. 2B), resembling localisation of the tight junction marker ZO-1 (Köppen et al., 2006). In contrast, cells of the DEL show cytosolic localisation of Pard3-GFP, with some local accumulations at the cortex (arrowheads, Fig. 2C). In contrast, Lin7c-RFP is distributed evenly in the nucleus and cytosol of both EVL and DEL cells (Fig. 2B–B″,C–C″,D–D″). These results show that i) Lin7c-RFP is not polarized in all epithelia, and ii) that the failure to become polarised is intrinsic to Lin7c itself and/or its interaction partners, rather than to a deficit in cell polarity, since Pard3 is localised in a polarised way in the EVL.

### Lin7c subcellular localisation reflects the refinement of retinal epithelial polarity

**Fig. 3.**
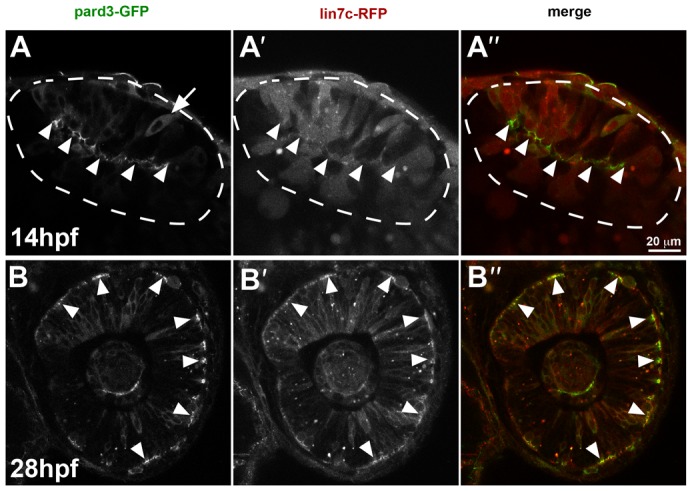
Lin7c-RFP subcellular localisation reflects temporal changes in retinal neuroepithelial polarity. (**A–A″**) In the optic vesicle (14 hpf, 10ss), Pard3-GFP is observed predominantly at the apical surface (arrowheads) of cells, except for a few cells, which show a cytoplasmic distribution (arrow) (A). Lin7c-RFP is cytosolic and not observed apically (A′). (**B–B″**) Cells of the retinal neuroepithelium (28 hpf) show apical localization of both Pard3-GFP (B) and Lin7c-RFP (B′). Scale bar: 20 µm.

The eye evaginates from the forebrain, forming the optic vesicle, which undergoes a series of very complex morphogenetic movements to give rise to the eye cup at 24 hpf (Schmitt and Dowling, 1994). Our results show that RFP-Lin7c is polarised in the retinal epithelium at the end of these processes (Fig. 1C). To determine if Lin7c polarisation coincides with general polarisation, we compared Lin7c-RFP and Pard3-GFP localisation at different stages of eye development. After formation of the optic vesicle, at 14 hpf, Pard3-GFP shows a clear apical localisation with only some protein remaining in the cytoplasm (Fig. 3A). In contrast, Lin7c-RFP is evenly distributed in the cytoplasm at this stage (Fig. 3A′). At 28 hpf both Pard3-GFP and Lin7c-RFP show a clear apical accumulation in cells of the retinal epithelium (Fig. 3B–B″). From this we conclude that i) the retinal epithelium is polarised at 14 hpf and can properly target apical proteins, such as Pard3, and ii) that Lin7c-RFP becomes polarised in the retinal epithelium as development proceeds. The behaviour of RFP-lin7c in the developing fish retina shows striking similarity to Lin7c localization in the developing rat neocortex: being diffusely distributed throughout the cell at early embryonic stages (E9), it becomes localized to the apical surface only later (Srinivasan et al., 2008). Therefore, fluorescently tagged Lin7c turns out to be an ideal tool to follow the refinement of epithelial polarity in vivo.

### Apical localisation of RFP-Lin7c is dependent on *ome/crb2a* and *nok/mpp5a* during eye formation

**Fig. 4.**
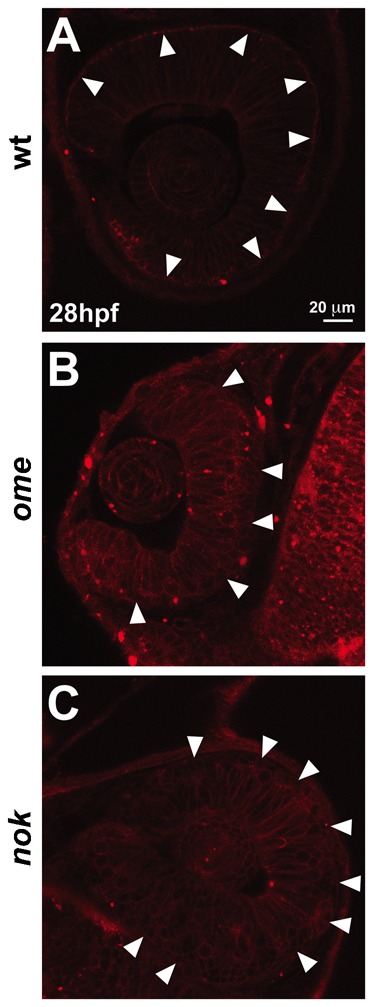
Nok and Ome are required for apical localisation of RFP-Lin7c. RFP-Lin7c sub-cellular localisation in the retinal neuroepithelium at 28 hpf. (**A**) In wild-type (wt) embryos, RFP-Lin7c is associated with the cell membranes, with a strong accumulation at the apical side (arrowheads). (**B**,**C**) RFP-Lin7c apical localisation is lost in *ome* and *nok* mutants (arrowheads). Note that in both mutants membrane association of RFP-Lin7c is maintained. Scale bar: 20 µm.

Both during *Drosophila* embryogenesis and during zebrafish retinal development, members of the Crb complex are required to ensure epithelial polarity and integrity. Nok and Ome are clearly localized apically in the retinal epithelium at 28 hpf (Wei and Malicki, 2002; Omori and Malicki, 2006), raising the question whether they are required for localizing RFP-lin7c. To address this question we made use of zebrafish embryos mutant for *nok* and *ome*. In both mutants, retinal polarity is disrupted at early stages (Malicki and Driever, 1999; Wei and Malicki, 2002). Apical accumulation of RFP-Lin7c in the retinal neuroepithelium is lost in both *nok* and *ome* mutants (Fig. 4), showing that Nok and Ome are required for proper apical localisation of Lin7c. Interestingly, RFP-Lin7c is associated with the plasma membranes in *ome* and *nok* mutants, suggesting that membrane recruitment of the initially cytosolic RFP-lin7c is independent of the Crb complex in the developing retina. These results show that RFP-Lin7c i) behaves as endogenous Lin7c in that it depends on Nok for apical localization (Yang et al., 2009), and ii) reflect a new Crb-independent step in polarity maturation.

Our results show a refinement of RFP-Lin7c localization in the developing retina: while initially cytosolic, RFP-Lin7c is recruited to the plasma membrane in a Crb-independent process, followed by apical enrichment in a Crb-dependent process. Future studies will elucidate the requirements for membrane recruitment of Lin7. In the mouse intestinal epithelium, Lin7C is associated with the baso-lateral membrane, a process that depends on CASK (calcium/calmodulin-dependent serine protein kinase), a scaffolding protein of the MAGUK family (Lozovatsky et al., 2009). Similarly, Lin-7C localization at the baso-lateral surface of Madine Darbine Canine Kidney (MDCK) cells and in renal epithelia requires its interaction with CASK (Straight et al., 2000). Since Pard3-GFP is apically localized prior to Lin7c-RFP localization, it is tempting to speculate that a hierarchy of polarity complexes governs refinement of polarity in the retinal neuroepithelium, similar to the developing blastoderm of the *Drosophila* embryo, where Bazooka/Par-3 localization precedes apical Sdt and Crb localization (Krahn et al., 2010), or in the zebrafish neural tube, where apical localization of N-cadherin and ZO-1 occurs before apical recruitment of Lin7c (Yang et al., 2009).

Lin7 not only exhibits various localisations in different cell types, but can also be found in different locations within the same cell. In mouse photoreceptor cells, for example, Lin7 is part of the Crb protein complex localised apically at the inner segment, apical to the outer limiting membrane, OLM, while it is part of a different complex at the photoreceptor synapse (Stöhr et al., 2005; Aartsen et al., 2006). It will be interesting to determine whether RFP-Lin7c is also recruited to the synaptic region in mature photoreceptor cells of zebrafish. To conclude, using fluorescently tagged-Lin7c will allow to study the dynamics of apical versus synaptic recruitment, and hence will provide insights into the mechanisms of polarity development in PRCs. Additionally, fluorescently tagged Lin7c is not only a useful tool to follow maturation of polarity in other zebrafish epithelia, but may also be used as a marker to follow changes occurring during pathological processes, such as metastasis, which has been associated with downregulation of Lin7c in some cases, for example in oral squamous cell carcinoma (OSCC) in human (Onda et al., 2007).

## Materials and Methods

### Fish maintenance and strains

Fish were maintained under standard conditions (Westerfield, 2000). Embryos were raised at 28.5°C in E3 medium and staged as previously described in hours post fertilization (hpf) (Kimmel et al., 1995). The following fish strains were used: wild-type AB, Tg(Ola.Actb:Hsa.HRAS-EGFP)^vu119^, made from a construct where the last 20 amino acids of c-Ha-Ras are fused to eGFP, targeting it to the plasma membrane under the control of the *Medaka* beta-actin promoter (Cooper et al., 2005), *nok^m520^* (Wei and Malicki, 2002) and *ome^m289^* (Omori and Malicki, 2006).

### DNA constructs, mRNA synthesis and injections

The lin7c fusion constructs were generated by cloning the PCR-amplified *lin7c* coding sequence into pCS2+N-mRFP or pCS2+mRFP-C vectors (Strzelecka et al., 2010). Capped mRNA was synthesized using mMESSAGE mMACHINE®SP6 Kit (Ambion) following the manufacturer's protocol. RNA was diluted to 1 µg/µl in distilled, sterile H_2_O and stored at −80°C. The amount of mRNA injected was estimated from the concentration and volume of a sphere of RNA solution (with 0.2% Phenol Red) injected into oil at the same pressure settings. For global or mosaic expression, RNA was injected in one blastomere of one-cell or 16/32-cell stage embryos, respectively.

### Live imaging

Embryos were treated with 0.2 mM Phenylthiourea (PTU) (Sigma–Aldrich) from 10 hpf onwards to prevent pigmentation. Prior to live imaging, embryos were anesthetized with 0.04% Ethyl 3-aminobenzoate methanesulfonate (MESAB) (Sigma–Aldrich). Embryos were mounted in 1.5% low melting agarose and medium containing PTU and MESAB was added. Imaging was performed in an LSM710 confocal with a 40× NA1.0 dipping lens (Carl Zeiss Microimaging). For co-localization studies, channels were acquired by sequential scanning. Image analysis and assembly was done with Fiji, Adobe Photoshop and Adobe Illustrator.
